# Non-invasive coronary wave intensity analysis

**DOI:** 10.1007/s10554-017-1185-0

**Published:** 2017-06-19

**Authors:** Christopher J. Broyd, Fausto Rigo, Justin Davies

**Affiliations:** 1 Imperial College London, London, UK; 2 Division of Cardiology, dell’Angelo Hospital, Mestre-Venice, Italy; 3 National Heart and Lung Institute, Hammersmith Hospital, Du Cane Road, London, W12 0HS UK

**Keywords:** Coronary physiology, Wave intensity analysis, Microcirculation

## Abstract

Wave intensity analysis is calculated from simultaneously acquired measures of pressure and flow. Its mathematical computation produces a profile that provides quantitative information on the energy exchange driving blood flow acceleration and deceleration. Within the coronary circulation it has proven most useful in describing the wave that originates from the myocardium and that is responsible for driving the majority of coronary flow, labelled the backward decompression wave. Whilst this wave has demonstrated valuable insights into the pathogenic processes of a number of disease states, its measurement is hampered by its invasive necessity. However, recent work has used transthoracic echocardiography and an established measures of central aortic pressure to produce coronary flow velocity and pressure waveforms respectively. This has allowed a non-invasive measure of coronary wave intensity analysis, and in particular the backward decompression wave, to be calculated. It is anticipated that this will allow this tool to become more applicable and widespread, ultimately moving it from the research to the clinical domain.

## Introduction

Wave intensity analysis was initially used in the field of gas dynamics but has now found marked applicability in assessing cardiovascular physiology. It is particularly useful in the coronary system as it can not only quantify the periodic forces acting within a single cardiac cycle but can also separate them according to their point of origin. Within the coronary circulation both the proximal (aortic) and distal (myocardial) arterial ends are potentially influential and wave-intensity analysis allows their individual contributions to be measured even when they occur simultaneously.

Wave-intensity is derived from simultaneously acquired pressure and flow velocity waveforms which can conveniently be measured using a dual-tipped Doppler- and pressure-sensor wire. Six waves are evident per cardiac cycle in both health and disease. The most clinically relevant wave has proven to be the backward decompression wave (BDW) which originates from the myocardium and due to its decompressive nature is responsible for drawing blood into the coronary arteries. This wave, which is formed by the diastolic elastic re-expansion of the microcirculation, has the most significant impact on coronary flow velocity. The BDW is therefore thought to provide information on the coupling between aorta and myocardium [1, 2].

Wave intensity in the coronary arteries has been investigated in a number of disease states including left ventricular hypertrophy, aortic stenosis, heart failure and ischemic heart disease to provide insightful, diagnostic and prognostic information on the function of the myocardium and microcirculation. Despite this, its clinical applicability is currently hampered by its need to be assessed using invasive tools. However, recent work by our group has expanded this technique into the non-invasive arena. As such, it has the potential to be applied to larger cohort based studies and transferred more broadly into the clinical environment.

## Mathematical concepts

A wave is a “disturbance that propagates in space and time” and this propagation involves the exchange of energy. In the cardiovascular system, this exchange is between blood’s kinetic energy and the potential energy in the walls of the elastic vessels. Waves can travel forward, from proximal to distal vessels, or backward, from distal to proximal vessels. They influence the medium in which they travel depending on whether they are a compressive or decompressive force and their results can therefore be appreciated as either causing acceleration or deceleration. It is important to realize that coincident waves with opposing effects will have a summative result so that if a forward acceleration wave meets a backward deceleration wave of the same magnitude there will be no net change in the velocity. However, despite this apparently static period on the velocity waveform, wave-intensity analysis is capable of identifying both waves.

Whilst the derivation of separated wave-intensity analysis is relatively complex, the fundamental definition of wave-intensity (I) is as the produce of the first derivative of pressure (dP) and flow (dU):

$$I = dP dU$$

Wave intensity has the units Wm^−2^. Its sign (positive or negative) conveys the direction of the dominant wave, positive values originating proximally and negative values originating distally. In order to control for the sampling frequency, the definition of wave intensity usually incorporates the sampling rate as:

$$I\text{'}=\left(\frac{dP}{dt}\right)\left(\frac{dU}{dt}\right)$$

The units are now Wm^−2^s^−1^.

Whilst the derivation of wave-intensity analysis is beyond the remit of this article [3], the formula for separated wave-intensity is relatively simple as shown:

$${I_ + } \equiv \frac{1}{{4\rho c}}{\left( {dP + \rho c dU} \right)^2}$$

$${I_ - } \equiv \frac{{ - 1}}{{4\rho c}}{\left( {dP - \rho c dU} \right)^2}$$

The only additional requirement for this formula is knowledge of local wavespeed (c) and the density of blood. The latter is taken as 1050 kg m^−3^ and the former calculated from the single-point wavespeed formula [4]:

$$c = \frac{1}{{\rho ~}}\sqrt {\frac{{\mathop \sum \nolimits^{} dP^{2} }}{{\mathop \sum \nolimits^{} dU^{2} }}}$$

Finally, wave intensity can be expressed in three ways: Firstly, the ‘peak wave intensity’ is defined as the maximum value of the wave intensity. Secondly, the ‘cumulative wave intensity’ is defined as the area under the wave intensity versus time curve. Thirdly, the recently-termed [5] ‘wave energy fraction’ is defined as the cumulative wave intensity for a particular wave divided by the integral of the wave intensity over the cardiac period.

## Coronary wave intensity analysis

**Fig. 1:**
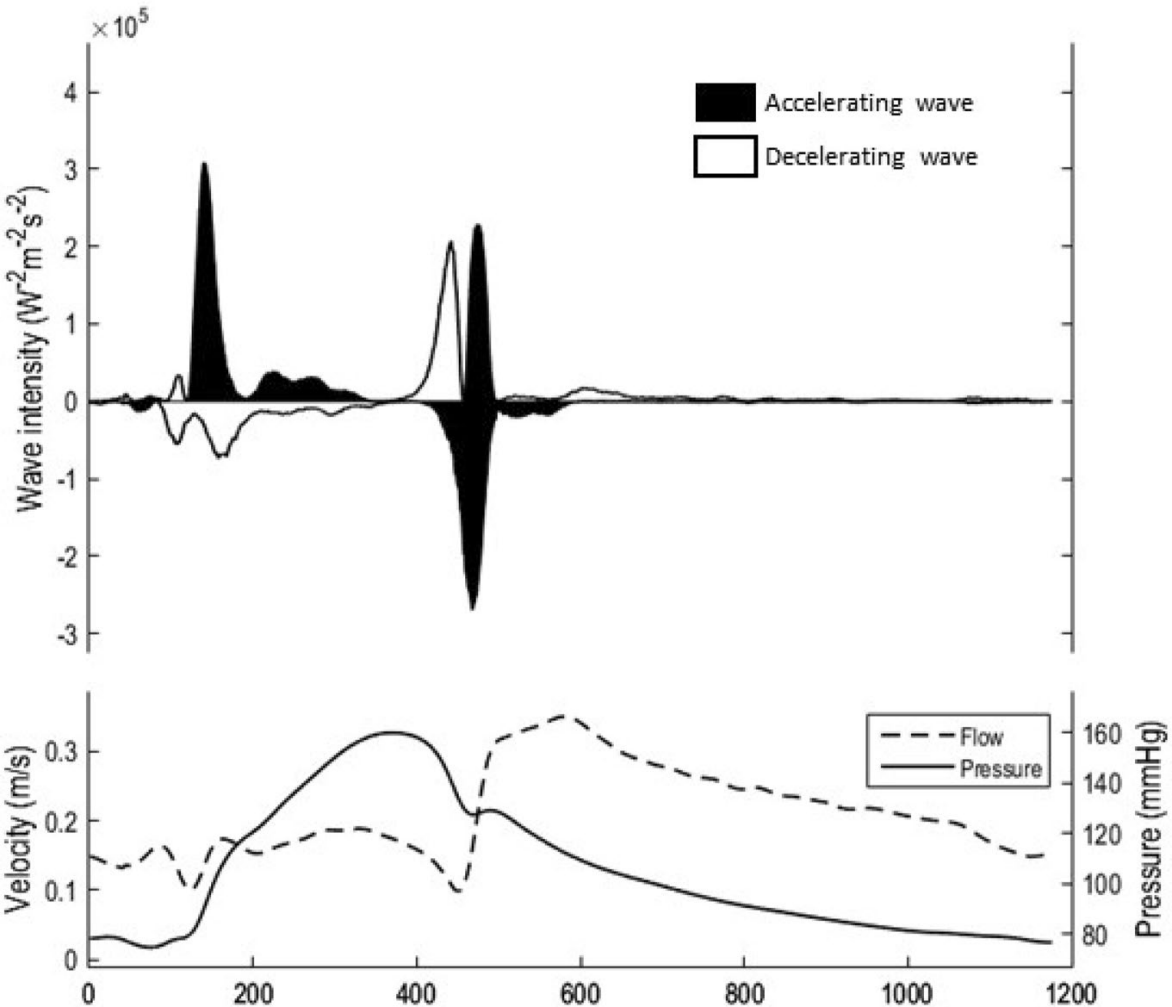
Pressure and flow recorded from the mid-LAD using a combowire (*lower panel*) is used to produce a wave-intensity signal (*upper panel*). Waves originating below the zero-line originate distally and above the line proximally. They are decompressive or compressive and therefore have either an accelerating or decelerating effect on coronary blood velocity

Six waves can be identified in heath and each has been ascribed to a mechanical part of the cardiac cycle [6]. Three waves originate proximally and three distally with variable compressing/decompressing and accelerating/decelerating effects (Fig. 1).

Of those waves that originate from the proximal (aortic) end, three are evident, described here from the onset of systole:

1. Forward compression wave (accelerating)—produced by contraction of the left ventricle with an open aortic valve. As blood is ejected into the aorta, the wave which originates in the left ventricle, is transmitted into the aorta and thus down the coronary artery in an antegrade direction.
2. Forward decompression wave (decelerating)—as systole ends, the slowing of ventricular contraction creates a suction effect in the aorta at the proximal end of the coronary artery.
3. Late forward compression wave (accelerating)—as the aortic valve closes a short-lived proximal-to-distal compression wave occurs.

Three distal (myocardial) waves are also evident, again described consecutively from early systole:

1. Early backward compression wave (deceleration)—prior to the aortic valve opening, the period of isovolaemic contraction results in compression of the intra-myocardial blood vessels which generates a distal-to-proximal compression wave.
2. Late backward compression wave (deceleration)—a second distal-to-proximal compressive wave is generated in early systole by the continuing compression of the microcirculation. Additionally, as the forward compression wave meets bifurcation sites or compressed mirovasculature, reflection of this wave occurs contributing to the late backward compression wave.
3. Backward decompression wave (acceleration)—this wave originates from the myocardium but is decompressive, therefore it causes an accelerative force to be applied to blood flow. This wave is created by the re-expansion of the compressed intra-myocardial blood vessels. To-date this is the most clinically relevant wave.

Of note, whilst the magnitude of the individual waves within each coronary artery is variable, the pattern of 6 waves is consistent and reflects the subtended muscle mass [7].

## Non-invasive coronary wave intensity analysis

**Fig. 2:**
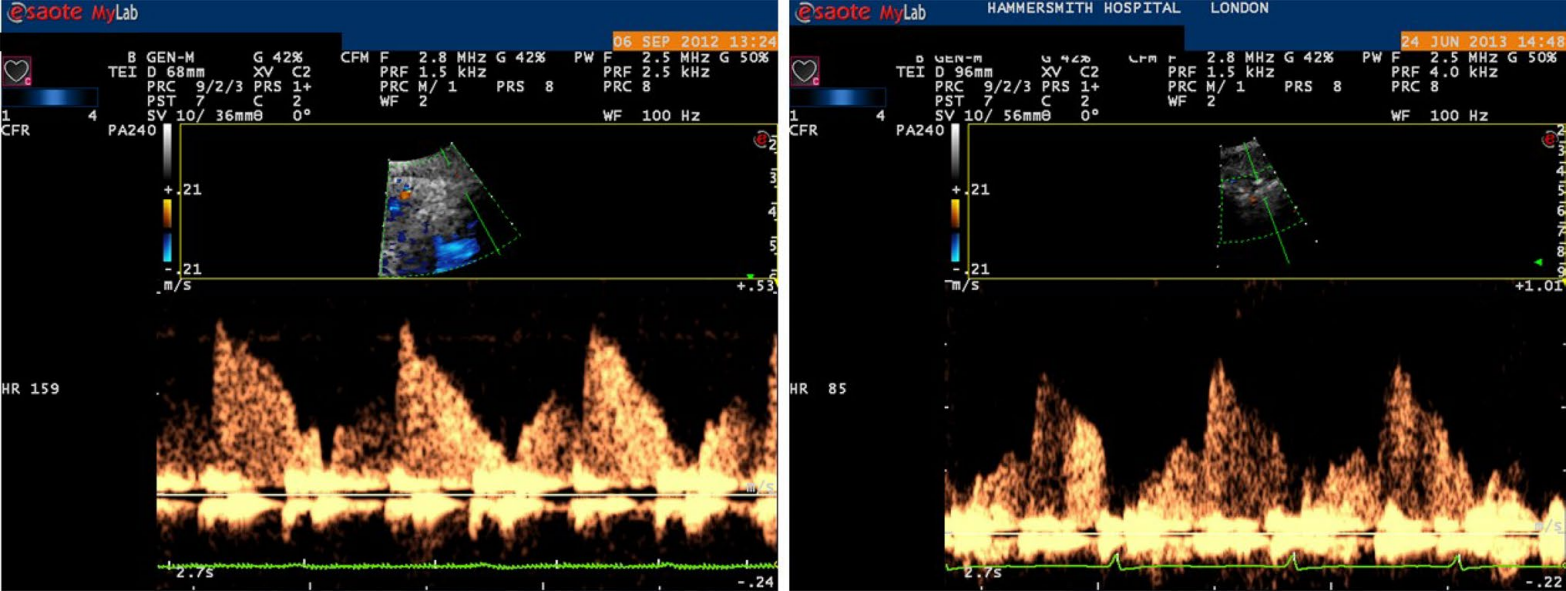
Example of non-invasively obtained coronary flow using transthoracic echocardiography

Thanks to the introduction of 2nd harmonic imaging and high frequency transducers, it has become possible to obtain a very accurate coronary flow envelope using echocardiography (Fig. 2). The left anterior descending artery is the easiest vessel to image but conventional probe positioning on the chest will not adequately demonstrate this vessel. Commencing in either a parasternal long axis or apical view the probe is moved across the chest to a modified parasternal view with septum maintained centrally. Machine parameters are altered in order to focus on the coronary artery [8]. Once visualized, pulse wave Doppler can be applied to sample flow (Fig. 2).

This measure shows equivalence with invasively derived measures [8–10]. This technique is now able to calculate coronary flow reserve and has been used to predict outcome in a variety of disease states [11–13]. Additionally, recognition of resting flow patterns, such as early systolic flow reversal or diastolic deceleration time has been applied to echocardiographically derived flow measures with some success [14]. However, the integration of flow velocity with pressure waveforms provides a much greater wealth of information. Accordingly, our group recently set out to construct a non-invasive measure of coronary wave intensity analysis.

In addition to echocardiographically derived flow a non-invasive measure of coronary pressure is also therefore required. Because reliable non-invasive measurements of central aortic pressure have been desirable for cardiovascular risk stratification a number of techniques exist. One option involves the application of a generalized transfer function to radial artery waveforms achieved from applanation tonometry. This is produced through averaging the individual functions calibrated to brachial cuff sphygmomanometer-measured BP; the transfer function is necessary to correct for pressure wave amplification in the upper limb [15, 16].

**Fig. 3:**
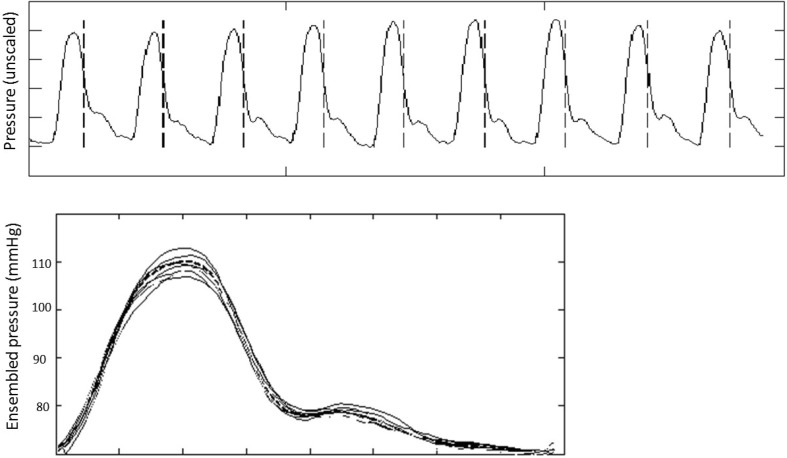
Central pressure obtained from the Pulsecor device (*upper panel*) and ensemble-averaged signal (*lower panel*). *Upper panel* raw data from the suprasystolic blood pressure system with the minimum dPdt for each cardiac cycle marked with* dashed vertical lines* and signals are ensembled according to these fiducial points. *Lower panel* ensemble average (*dashed line*) with the individual aligned waveforms (*solid lines*). Reproduced from Broyd et al. [5]

An alternate option is through use of suprasystolic brachial pressure waveform interpretation. This system estimates central pressures from brachial cuff pressure fluctuations by inflating to suprasystolic levels (approximately 30 mmHg above systolic pressure) and occluding the brachial artery. Intra-arterial pressure waves impinging on the occluded artery transfer part of their energy to the surrounding upper arm tissue and then to the cuff and can be directly related to the intra-arterial pressure oscillations. This also means there are no confounding waves reflected distal to the point of measurement. A time-domain representation of pressure wave reflection within a uniform closed tube has been established and applied to the cuff-sensed pressure to produce central aortic pressure. Measuring central pressure in this method has been demonstrated to be comparable to ‘gold-standard’ invasive data [17, 18] (Fig. 3).

**Fig. 4:**
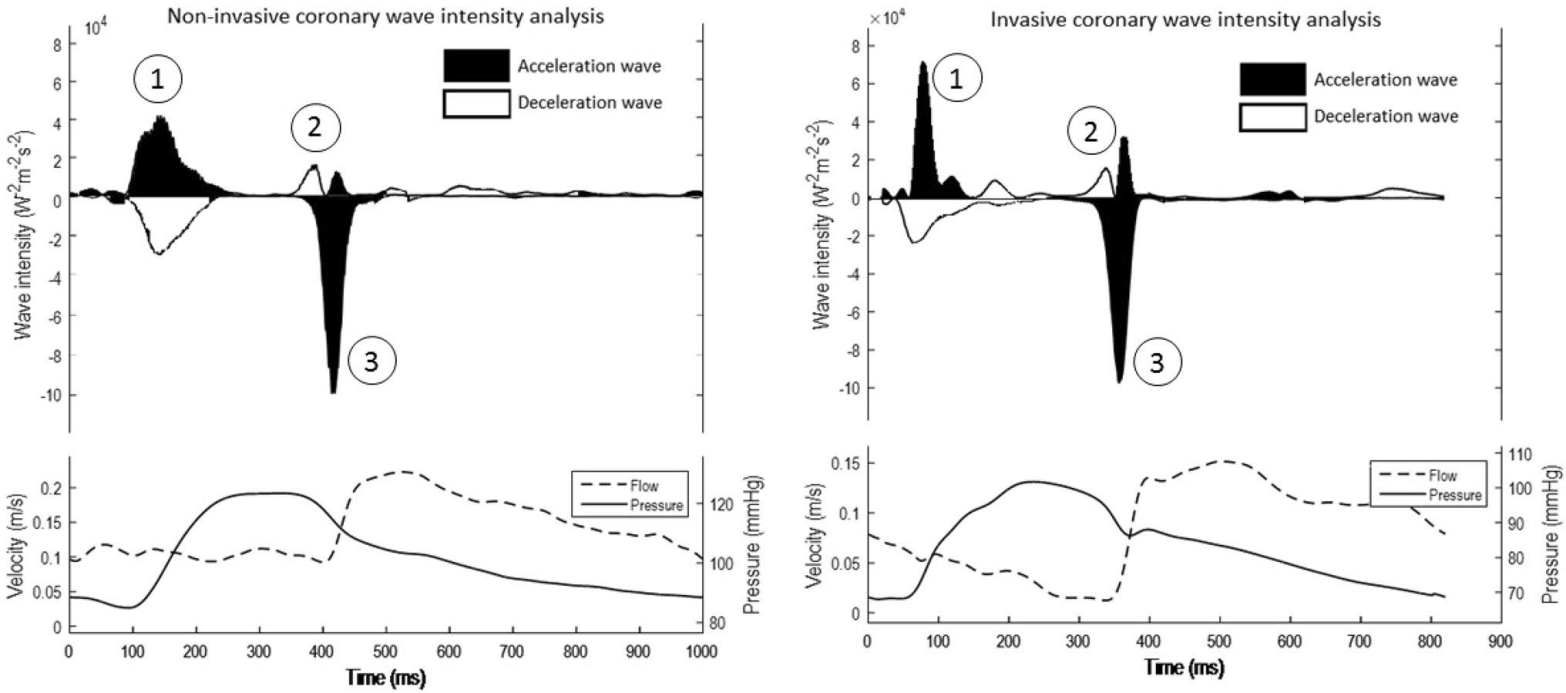
Non-invasive (*left*) and invasive (*right*) coronary wave-intensity analysis achieved from the same patient measured within the mid-LAD. Three waves are highlighted: the forward compression wave (*1*), the forward decompression wave (*2*) and the backward decompression wave (*3*). Reproduced from *Broyd et al*. [5]

**Fig. 5:**
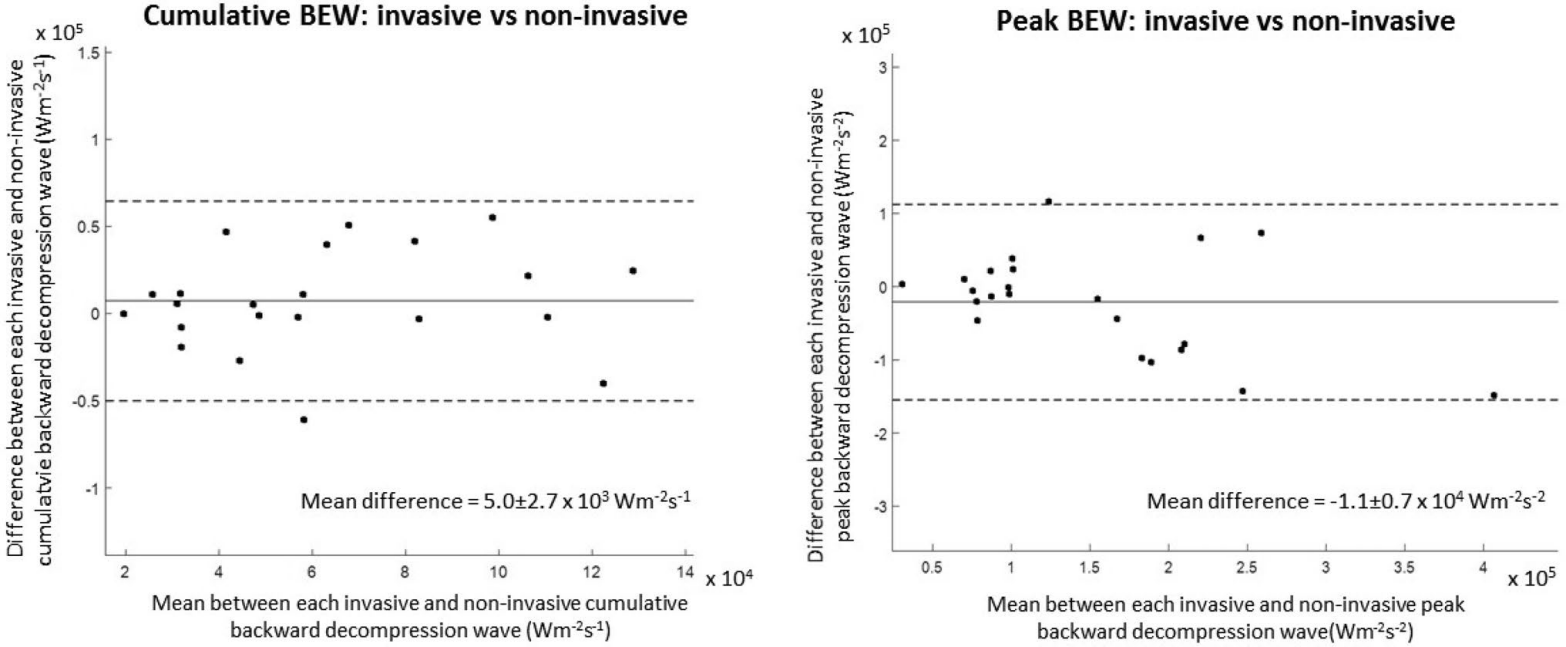
Bland Altman plot of invasive versus non-invasive backward decompression wave: peak (*left*) and cumulative (*right*), *Solid horizontal line* represents mean difference and *dashed lines* the limit of agreement (±1.96 × SD). Reproduced from Broyd et al. [5]

Using this latter system, in conjunction with simultaneously measured coronary flow, non-invasive coronary wave-intensity analysis has recently been validated [5] and the same repeating patterns of waves can be appreciated within the cardiac cycle (Fig. 4). Measuring the backward decompression wave through this approach has been shown to be accurate when compared to invasive data gathered from the same individual. Because of the technicalities in preserving this wave as accurately as possible the other waves within the cardiac cycle were significantly underestimated (Fig. 5).

**Fig. 6:**
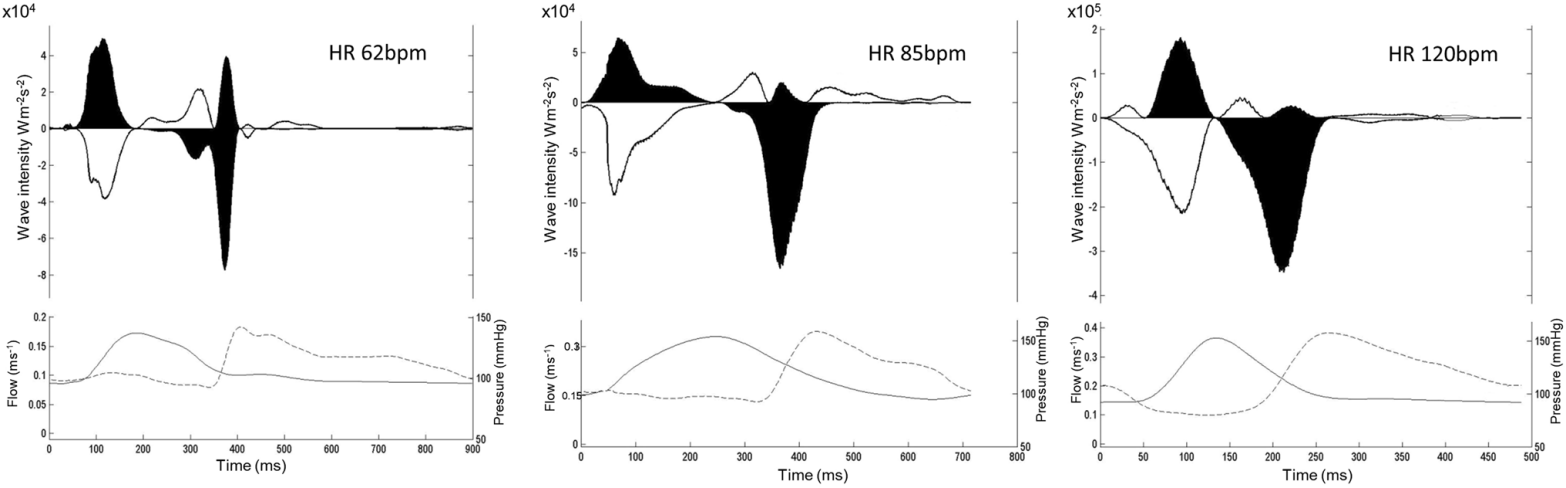
Coronary flow assessment and non-invasive wave-intensity analysis at increasing heart rates. Rest is displayed on the left, mid-exertion centrally and maximum exertion on the right. With exercise and a resultant increasing heart rate a progressive increase is seen in the in size of the cumulative and peak backward decompression wave. This reflects a greater ‘suction’ effect from the myocardium resulting in higher coronary flow rates per cardiac cycle. *HR* heart rate. Reproduced from Broyd et al. [5]

**Fig. 7:**
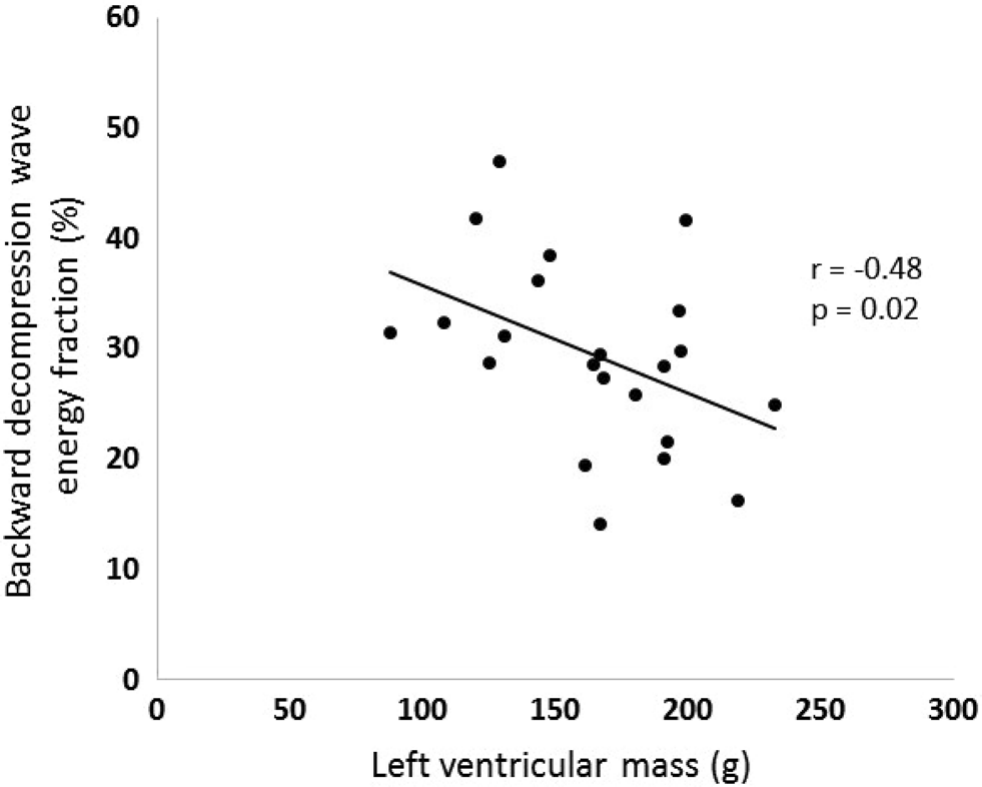
Scatterplot showing the relationship between the non-invasive backward decompression wave energy fraction and left ventricular mass. The *solid line* represents the regression line and Pearson’s correlation coefficient is shown. Reproduced from Broyd et al. [5]

Furthermore, wave-intensity analysis produced through this approach responds appropriately in physiological and pathological settings. Firstly, using a static exercise bike the backward decompression wave is seen to increase in magnitude as has been demonstrated invasively [2] (Fig. 6). Secondly, with increasing left ventricular mass the backward decompression wave becomes attenuated (Fig. 7). Again, this is similar to the relationship between left ventricular wall thickness and the backward decompression wave seen invasively obtained data [6].

## Potential applications

With the transition of coronary wave-intensity analysis into the non-invasive environment, a vast number of clinical investigative opportunities are opened. As it can be measured in the majority of patients, carries no risk and requires no pharmacological agents its key feature is in its ability to perform serial measurements.

Our group has previously shown that patients with aortic stenosis have a strikingly abnormal wave-intensity profile that normalises immediately following valve implantation [1]. Using non-invasively derived measures of wave intensity may permit a further measure of myocardial burden to be estimated in patients with mild or moderate aortic stenosis, or in those with low-flow low-gradient valves, and thus aid the timing of intervention.

**Fig. 8:**
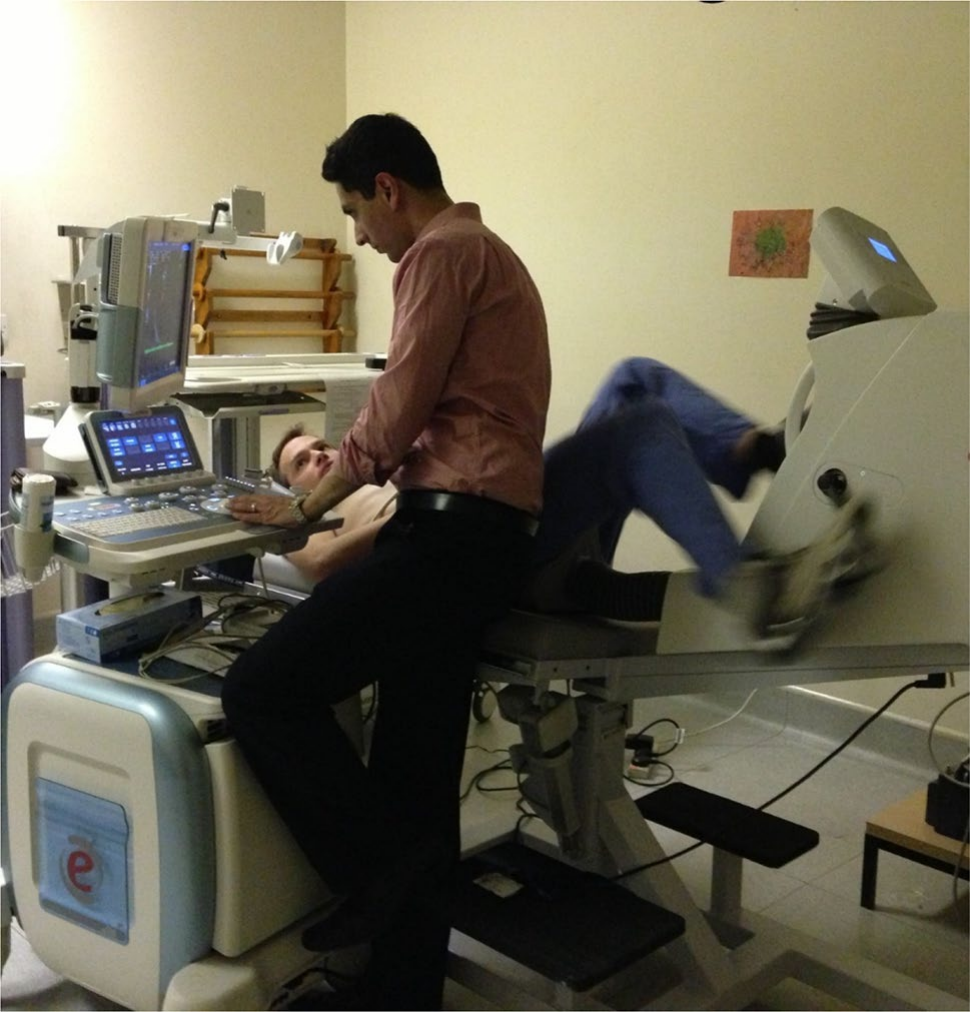
Exercise coronary wave-intensity analysis performed non-invasively. Using a static exercise bike exercise coronary wave-intensity can be performed non-invasively

With exercise (Fig. 8) or pharmacological stress, valve disorders (particularly aortic stenosis) could be serially assessed during stress to allow timing of intervention, looking for a potential ‘tipping point’ when myocardial ischemia begins to dominate [19]. Additionally, the ability of wave-intensity analysis to recognize the subtle differences in biventricular pacing regimens has been highlighted [20]. To optimize these devices non-invasively according to coronary wave-intensity analysis obtained through this approach would be highly desirable and now potentially possible.

Its potential use as a pre-screening tool is also now obvious. Because wave-intensity analysis is able to recognize subtle resting abnormalities in myocardial function [21] it may have potential in patients with risk factors for cardiovascular disease in order to stratify their pharmacological therapy. In those at risk with an abnormal resting wave-intensity profile, treatment could be instigated early and followed to ensure normalization.

Finally, in the area of unstable coronary disease, wave-intensity analysis has been used to offer prognostication in patients who suffered from non-ST elevation myocardial infarction [22]. Of note, the BDW in the infarct related artery (measured within 48 h of the event) correlates with both biochemical- and MRI-markers of infarct severity. Therefore, non-invasive coronary wave-intensity could be a convenient tool for prognostication after a myocardial infarction.

Despite this, further investigative work is required in this field. Particularly, the accurate non-invasive measurement of the other waves within the cardiac cycle is likely to be important for future applications. Furthermore, its applicability may be limited to those without obstructive coronary disease. However, it may still provide useful large-based cohort data or be useful intra-patient follow up work. Finally, its reproducibilty on a larger scale will be important to establish for work involving long-term patient follow-up.

## Conclusion

Coronary wave-intensity analysis is a powerful tool in assessing the coupling between aorta and myocardium. Despite the wealth of information it can provide, it has been is limited by its invasive necessity. However, recent efforts to construct non-invasive coronary wave-intensity analysis have proved successful which may vastly increase its applicability. Its potential use as a pre-clinical screening tool and as an assessment technique for established disease is now emerging.
